# Modeling early events in *Francisella tularensis* pathogenesis

**DOI:** 10.3389/fcimb.2014.00169

**Published:** 2014-12-11

**Authors:** Joseph J. Gillard, Thomas R. Laws, Grant Lythe, Carmen Molina-París

**Affiliations:** ^1^Defence Science and Technology LaboratoryPorton Down, Salisbury, UK; ^2^Department of Applied Mathematics, School of Mathematics, University of LeedsLeeds, UK

**Keywords:** *Francisella tularensis*, stochastic modeling, pathogenesis, object-oriented modeling, intracellular infection, compartmental models, macrophages, lung diseases

## Abstract

Computational models can provide valuable insights into the mechanisms of infection and be used as investigative tools to support development of medical treatments. We develop a stochastic, within-host, computational model of the infection process in the BALB/c mouse, following inhalational exposure to *Francisella tularensis* SCHU S4. The model is mechanistic and governed by a small number of experimentally verifiable parameters. Given an initial dose, the model generates bacterial load profiles corresponding to those produced experimentally, with a doubling time of approximately 5 h during the first 48 h of infection. Analytical approximations for the mean number of bacteria in phagosomes and cytosols for the first 24 h post-infection are derived and used to verify the stochastic model. In our description of the dynamics of macrophage infection, the number of bacteria released per rupturing macrophage is a geometrically-distributed random variable. When combined with doubling time, this provides a distribution for the time taken for infected macrophages to rupture and release their intracellular bacteria. The mean and variance of these distributions are determined by model parameters with a precise biological interpretation, providing new mechanistic insights into the determinants of immune and bacterial kinetics. Insights into the dynamics of macrophage suppression and activation gained by the model can be used to explore the potential benefits of interventions that stimulate macrophage activation.

## 1. Introduction

*Francisella tularensis* is a gram-negative bacterium that may be inhaled in an aerosol, resulting in respiratory or pneumonic tularemia (Oyston et al., 2004; Larsson et al., 2005; Oyston, 2008). Of its four subspecies, *F. tularensis* subspecies *tularensis* (type A) is the most lethal for humans, hence its designation as a category A biothreat agent by the Centers for Disease Control and Prevention (CDC). Much of the information describing its pathogenesis has been compiled using an attenuated type B strain, known as live vaccine strain (LVS) (Fortier et al., 1991; Ellis et al., 2002; Cole et al., 2011). However, in this paper we are concerned exclusively with *F. tularensis* type A, strain SCHU S4, which will be referred to below simply as *F. tularensis*.

*F. tularensis* is able to subvert, resist, or evade killing by antimicrobial defenses (Bosio et al., 2007; Jones et al., 2012). It enters alveolar macrophages (Ellis et al., 2002; Clemens et al., 2005; Hall et al., 2008; Straskova and Stulik, 2012) and dendritic cells (DCs) without inducing their classical activation (Mosser, 2003) or the release of pro-inflammatory cytokines. It is phagocytosed by alveolar macrophages, but is able to survive and escape from the phagosome to the cytosol in less than 1 h (Golovliov et al., 2003; Jones et al., 2012). After multiple rounds of division in the cytosol, the high bacterial load eventually causes the host macrophage to rupture and die, releasing many bacteria (Cowley and Elkins, 2011).

By entering macrophages without alerting the innate immune system, *F. tularensis* gains time for an initial growth of its population by replication in their hosts' cytosols (Polsinelli et al., 1994). The typical number of bacteria released from a ruptured macrophage, initially infected by a single bacterium, is estimated to be more than 100 (Wood et al., 2014). Further time is gained by active suppression of the inflammatory response to the debris from cell death. Infected macrophages and DCs display diminished responsiveness to lipopolysaccharide (LPS) (Telepnev et al., 2003; Bosio et al., 2007). Despite rapid replication of bacteria and rupture of host macrophages, *F. tularensis* does not elicit the typical pro-inflammatory responses associated with acute pulmonary bacterial infections within the first 48 h of infection, consistent with the hypothesis that *F. tularensis* induces local and systemic production of the transforming growth factor TGF-β (Bosio et al., 2007; Hall et al., 2008). Increased TGF-β levels have been found in the lungs and spleen of SCHU S4-infected mice compared with uninfected controls, 24 h post-infection (Bosio et al., 2007).

Because *F. tularensis* prevents immune recognition and the production of pro-inflammatory cytokines for up to 72 h post-infection (Jones et al., 2012), the subsequent response is hypercytokinetic and often fatal (Cowley and Elkins, 2011). Damage-associated molecular patterns (DAMP), such as the high-mobility group protein B1 (HMGB1), are detected at above normal levels in blood serum only after 72 h post-infection (D'Elia et al., 2013). Treatment of mice with anti-HMGB1 antibody causes a more effective immune response, characterized by increased levels of the interferon IFN-γ, which can widen the window of opportunity for antibiotic therapy (D'Elia et al., 2013).

Several notable examples of within-host mathematical models of infection have been published. For instance, in the context of *Mycobacterium tuberculosis* infection, Day et al. (2009) have considered the balance between populations of classically and alternatively activated macrophages (Gordon, 2003; Gordon and Martinez, 2010; Mattila et al., 2013). Their mathematical model, a system of ordinary differential equations (ODEs), is based on the two-compartment model (lung and lymph node) of Marino and Kirschner (2004). A hybrid model of *M. tuberculosis* that is agent-based in the lung compartment and a system of ODEs in the lymph node compartment has also been developed (Marino et al., 2011). Day et al. (2011) developed a two-compartment ODE model of host response to inhalation anthrax, while the deterministic computational model of Gutting (2014), that describes the bacterial kinetics of inhalational anthrax in New Zealand white rabbits, is a physiological-based bio-kinetic model in which one compartment is the lumen of the airways and the other the rabbit body. A two-compartment model, with movement of cells on a a two-dimensional lattice, has been developed by Attie and Daefler (2013). However, there are no prior examples of mechanistic computational models of *F. tularensis* SCHU S4 infection that have been developed for the explicit purpose of supporting the investigation and development of medical treatments.

Research into the development of treatments for *F. tularensis* infection revolves around the use of validated animal models to gain understanding of the mechanisms of pathogenesis and host response and to explore potential targets for intervention. The aim of the present study is to develop a computational model based on the BALB/c mouse model of inhalational SCHU S4 infection, that can be used as an investigatory tool to support experimentalists. Such a model must represent the key processes mechanistically; be determined by biologically relevant and measurable parameters; accurately simulate bacterial growth and proliferation, as observed *in vivo*, and offer the facility to represent medical interventions explicitly.

We present a stochastic model of *F. tularensis* SCHU S4 infection, with an object-oriented design that facilitates the addition of further levels of complexity in the future. The model includes bacterial replication in macrophages and three spatial compartments for which experimental results have been reported by D'Elia et al. (2013). We model the immune subversion tactics employed by *F. tularensis* during infection ensuring that, even after phagosomal escape, infected macrophages are in a deactivated state in which they are not able to induce inflammatory responses (Gordon, 2003; Mantovani et al., 2004; Bosio et al., 2007; Dai et al., 2013; Gillette et al., 2014; Martinez and Gordon, 2014). We shall refer to this macrophage state as “suppressed.” While macrophages exhibit a continuum of activation states (Mosser and Edwards, 2008), in our computational model we restrict attention to the most pertinent states, wherein macrophages become classically activated either by the effect of pro-inflammatory signals or in the presence of sufficient concentrations of IFN-γ. Thus, macrophages are represented broadly as resting, suppressed or activated. For this paper we consider in detail the first 48 h of infection and focus principally on events in the lung compartment. Immune response is dominated by resident macrophages during this early phase, therefore these phagocytes are considered primarily.

By being able to describe the pathogenesis computationally, we can gain insight into processes that are not necessarily accessible though experimental means. Ultimately, this work is a step towards a capability for conducting *in silico* investigations to help design *in vivo* experiments for evaluating candidate therapeutics for highly dangerous pathogens.

## 2. Materials and methods

### 2.1. Model description

In this section the development of a computational model of the early stages of *F. tularensis* infection, following aerosol exposure, is presented. The simplest stochastic models of cell populations are birth-and-death processes (Taylor and Karlin, 1998; Stirzaker, 2005; Lythe and Molina-París, 2011), where the size of the population changes by one cell at a time, due to the death or division of one of the cells in the population. Such models can be extended to multi-dimensional Markov processes, where the variables are the numbers of cells in distinct populations (Wood et al., 2014). Here, we maintain the framework of evolution of the system by a series of discrete events, extending the description of the population by giving each macrophage four attributes: a spatial location, a state of activation, a number of phagosomal bacteria, and a number of cytosolic bacteria. Events are no longer restricted to birth and death of cells; they affect the number, or the attributes, of cells of different types. The prescription of the mathematical model is an enumeration of the possible events and how their rates depend on parameters, and on the current state of the system. Given the parameters and their values, numerical solutions are generated using the Gillespie algorithm (Gillespie, 2007). Code for the model is included as supplementary material.

For comparison with experimental results (D'Elia et al., 2013), the spatial compartments we consider are lung, spleen and liver. Free bacteria suffer one of three fates: phagocytosis, migration or death. Migration between compartments is via the blood to a destination chosen randomly according to relative probabilities that are proportional to the actual weights of the organs. The host phagocytic cells, initial targets of the infection in the lung, are believed to be macrophages (Cowley and Elkins, 2011). Other types of cells are expected to act as hosts in other parts of the body but, for the purposes of this study, they will be referred to as macrophages. Similarly, at a later stage of infection, new phagocytes will migrate to the infected organs, but in this study the number of phagocytic cells only changes due to the rupture and death of infected macrophages.

### 2.2. Representation of macrophages

Macrophages are modeled as individual computational objects that possess the following attributes, which change during the infection process:

1. A spatial location, *l*: either lung, liver or spleen.
2. An integer number of bacteria in phagosomes, *b*. Initially 0, this attribute is set to 1 if a bacterium enters the macrophage. Values of *b* greater than 1 are allowed, but are rare in the first 48 h post-infection.
3. An integer number of bacteria in the cytosol, *c*, initially 0. When a bacterium escapes from a phagosome, the corresponding values of *b* and *c* are decreased and increased by 1, respectively. Each bacterial replication event, that occurs with rate β per cytosolic bacterium, increases *c* by 1.
4. A state of activation, *a* initially equal to 0 (corresponding to a resting state). Ingestion of a bacterium, or the effect of TGF-β, causes the macrophage to be put into a suppressed (or unresponsive) state, *a* = −1.

### 2.3. Modeling early stages of pathogenesis in the lungs

**Figure 1:**
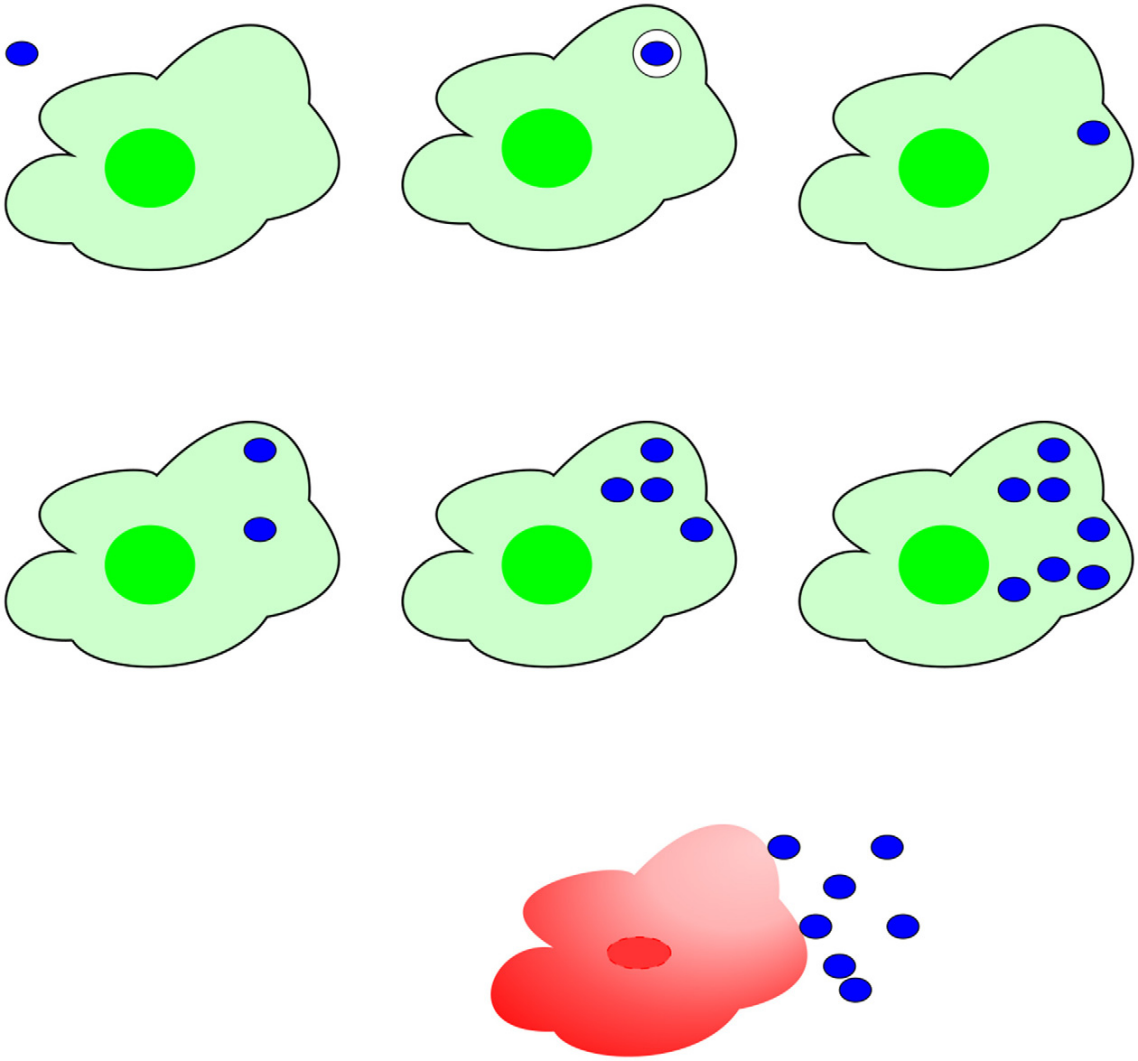
**Mechanism of *F. tularensis* pathogenesis**. **Top line:** A bacterium (blue), ingested by a macrophage (green), escapes from the phagosome to the cytosol. **Central line:** In the cytosol, bacteria proliferate, eventually **(bottom line)** provoking rupture and death of the macrophage and release of a large number of bacteria.

The basic dynamics of *F. tularensis* infection are illustrated in Figure 1. The mechanics of deposition in the alveolar space, which precedes infection, are outside the scope of our model. Therefore, the assumed initial state of the system at time *t* = 0, is that a dose of *N* free bacteria is located in the alveolar spaces, in proximity of *M* resting macrophages. Macrophage infection and bacterial replication then take place according to the following rules:

1. Macrophages internalize bacteria into a phagosome with rate ρ.
2. Free bacteria die with rate μ.
3. Phagocytosed bacteria escape from phagosome to cytosol with rate ϕ.
4. In the cytosol, bacteria divide with rate β.
5. Macrophages rupture and die, releasing their bacteria, with rate equal to δ multiplied by the number of bacteria in their cytosol.
6. Free bacteria leave the lung, with rate γ, and migrate to other parts of the body.
7. Macrophages change their state of activation, to a suppressed (or unresponsive) state with rate ν, or to the classically activated state with rate determined by the IFN-γ concentration, *G*(*t*).

### 2.4. Parametrization of the model

Parameter values were obtained from experimental literature and are summarized as follows:

- The initial number of macrophages in the alveolar space, where the initial dose is assumed to come to rest after inhalation, is typically *M* = 10^4^ (Condos et al., 1998; Marino and Kirschner, 2004). The rate of phagocytosis per macrophage, ρ, is taken to be 0.01 h^−1^ (Marino and Kirschner, 2004).
- The death rate of free bacteria is set to μ = 0.01 h^−1^ (Lowrie et al., 1979).
- The rate ϕ = 2 h^−1^ corresponds to a mean escape time of 30 min (Jones et al., 2012).
- The rate β = 0.15 h^−1^ corresponds to a mean division time less than 10 h (Lowrie et al., 1979; Jones et al., 2012).
- The rate δ is set to 0.001 h^−1^ (Marino and Kirschner, 2004).
- The migration rate is set to γ = 0.1 h^−1^ (Day et al., 2011; Ganusov and Auerbach, 2014).
- The rate ν is set to 0.01 h^−1^ (Day et al., 2011).

### 2.5. Computational methods

In the stochastic model of the mechanism of *F. tularensis* infection, individual host phagocytes and *F. tularensis* bacteria are represented. Each interaction between bacteria and host cells is considered explicitly, using the Gillespie stochastic simulation algorithm (Gillespie, 2007). The characteristic property of the Gillespie algorithm is that two random variables are drawn at each step. The first, uniformly-distributed in the interval (0,1), determines which event occurs and the second, exponentially-distributed, determines the length of time elapsed.

**Figure 2:**
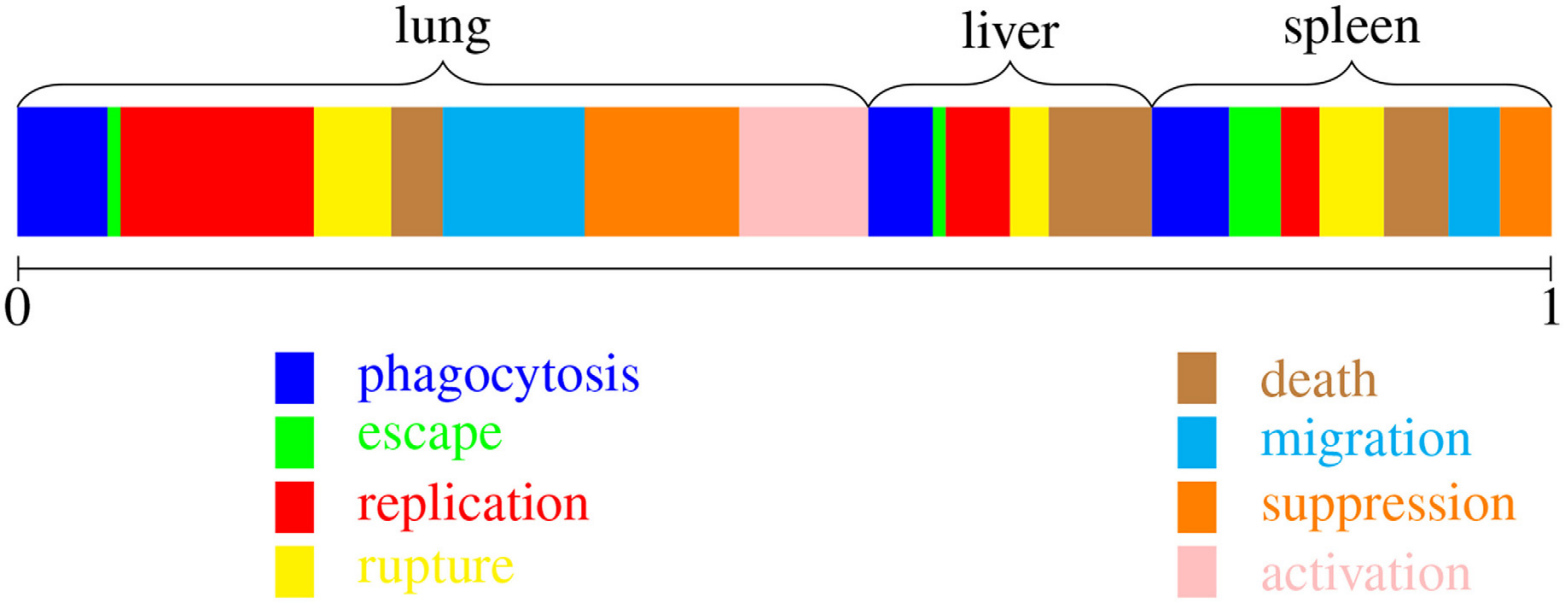
**Implementing the Gillespie algorithm**. At each step, one type of event is chosen, with probabilities weighted by the corresponding rates. There are twenty-four possibilities, corresponding to one of the colors and one of the three spatial locations.

In our model, with large numbers of computational objects representing bacteria and macrophages, we determine which event occurs at each step as follows. The unit interval is divided into sub-intervals represented in Figure 2; each sub-interval corresponds to one type of event. Here, there are eight types of event, as described above, in three spatial compartments, twenty four in total. At each step in the simulation, the probability that a given event is the next to occur is the width of the corresponding sub-interval.

The widths represented in Figure 2 are relative “total rates.” That is, they are summed over all the bacteria and macrophages capable of participating in the corresponding “reaction” or event. These rates depend on the current state of the system and are calculated at each step as follows. Let

- *b*(*t*) be the number of free bacteria,
- *m*_*r*_(*t*), *m*_*s*_(*t*) and *m*_*a*_(*t*) be the numbers of resting, suppressed and activated macrophages,
- *p*(*t*) be the total number of bacteria in phagosomes,
- *c*(*t*) be the total number of bacteria in cytosols,

in a chosen spatial location. Then the rates are computed as follows:

1. Phagocytosis, total rate = ρ *b*(*t*) [*m_r_*(*t*) + *m_a_*(*t*) + *m_s_*(*t*)].
2. Death of free bacteria, total rate = μ *b*(*t*).
3. Bacterial escape from phagosome to cytosol, total rate = ϕ *p*(*t*).
4. Division of bacteria in the cytosol, total rate = β *c*(*t*).
5. Macrophage rupture and death, total rate = δ *c*(*t*).
6. Migration of free bacteria, total rate = γ *b*(*t*). The destination is selected from the three possibilities, with relative weights: lung 0.2, liver 1.0, and spleen 0.1.
7. Suppression of resting macrophages by TGF-β, total rate = ν *m_s_*(*t*).
8. Activation of resting macrophages by IFN-γ, total rate = ν if *G*(*t*) > 100.

Once a type of event is chosen, it is also necessary to select which macrophage or bacterium will participate. For example, if the chosen event is phagocytosis in the lung, then one of the resting, suppressed or activated macrophages in the lung is selected (at random). To complete one step of the algorithm, *G*(*t*) is updated in each of the three compartments, according to $\frac{\text{d}}{\text{d}t}G=m_{a}(t)$.

Multiple realizations are run, with the initial number of *F. tularensis* bacteria chosen from a Poisson distribution with mean dose *N*. In this way, we can estimate the variation from experiment to experiment. The exact Gillespie stochastic simulation algorithm is practical for the first 2 days post-infection. Thereafter, the rapid increase in bacterial load produces numbers of cells that require tau-leaping methods (Tian and Burrage, 2004; Márquez-Lago and Burrage, 2007).

## 3. Results

### 3.1. Bacterial growth rate and doubling time

The computational model was used to simulate *F. tularensis* growth *in vivo* in the lungs of BALB/c mice for the first 48 h after exposure. For each of 100 runs, a starting dose was drawn from a Poisson distribution with mean 100 bacteria. Based on the mechanisms described in the previous section, each run produced a bacterial load profile for the lung compartment. A mean bacterial growth rate for the simulations was calculated as the mean of the gradient coefficients in the linear regression of each bacterial load profile (transformed to the logarithm base 10) against time. For comparison, growth rates of bacteria in BALB/c lungs were calculated from experiments published in D'Elia et al. (2013) and from unpublished data donated as a kin gift from R. Lukaszeswki. Inclusion criteria were that only time points between 0 and 48 h were used, there was a known challenge with strain SCHU S4 and mice were challenged via the intranasal route or the aerosol route.

**Figure 3:**
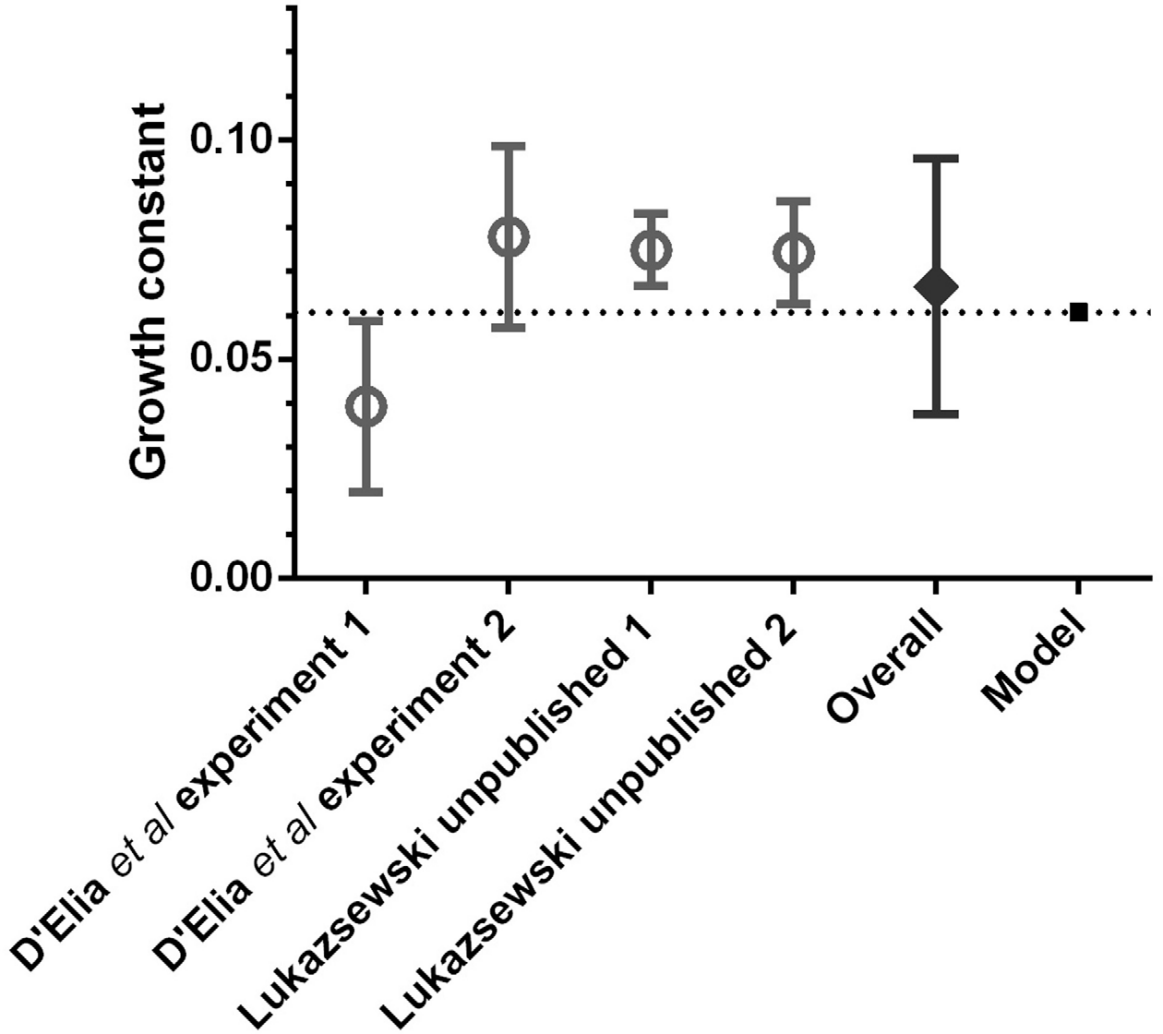
**Comparison of pulmonary bacterial growth rate predicted by the computational model (mean of 100 runs), with the *in vivo* bacterial growth rates observed in BALB/c mice after pulmonary infection with *F. tularensis*, during the first 48 h post-exposure**.

The comparison between experimental data and the model is shown in Figure 3. Error bars on the experimental growth rates show the 95% confidence limits of the parameter estimates. The “Overall” growth constant was calculated by taking the mean and 95% confidence intervals of the parameter estimates. Confidence intervals for the growth rate predicted by the model are not displayed, since the variability in bacterial loads between simulation runs is insignificant by the 48 h time-point, as compared with the large variability of experimental data. This is an artifact of using the same model parameters for each model run. Future work will explore how probability distributions may be used as model inputs, in order to simulate intra-subject variability more realistically.

This comparison with experimental data serves as a verification of the model mechanisms and a validation of its output. Furthermore, the value of the computational model growth constant for the first 48 h displayed in Figure 3 is 0.0607 h^−1^, leading to a doubling time of 5 h, which corresponds with the findings of Attie and Daefler (2013) and references therein. Therefore, the computational model predicts bacterial growth in the lungs accurately for the early stages of infection. Since the model is governed by a small number of experimentally verifiable parameters, this opens up the possibility of using the model as a theoretical tool to investigate, *in silico*, the required efficacy of therapeutic interventions that modify these parameters in order to reduce bacterial growth.

### 3.2. Bacterial dynamics of the first 24 h

*F. tularensis* is highly infectious and aerosolisable, capable of causing a debilitating or fatal disease with doses as low as 25 colony-forming units (Oyston et al., 2004). In this section, we investigate the bacterial dynamics following a relatively low initial dose of *F. tularensis* such that macrophages vastly outnumber bacteria in the region of the lung where the bacteria come to rest (*M* ≫ *N*). In this case, all bacteria are phagocytosed in a few minutes, and it is improbable for any macrophage to ingest more than one bacterium.

To understand the earliest post-infection phase, after the initial uptake of bacteria and before any macrophages rupture and die, it is illuminating to define two mean quantities. Let *P*_0_(*t*) be the mean number of bacteria that are in macrophage phagosomes at time *t*. Let *C*_0_(*t*) be the mean number of bacteria, and their descendants, in macrophage cytosols at time *t*, assuming that no rupture and death events have yet occurred. These mean quantities satisfy the following ODEs:

(1a) $$\frac{\text{d}}{\text{d}t}P_{0}=-\phi P_{0}\;,$$

(1b) $$\frac{\text{d}}{\text{d}t}C_{0}=\phi P_{0}+\beta C_{0}\;.$$

With the initial conditions *P*_0_(0) = *N* and *C*_0_(0) = 0, the solution is

(2a) $$P_{0}(t)=N{\text{e}}^{-\phi t}\;,$$

(2b) $$C_{0}(t)=N^{\prime }\left({\text{e}}^{\beta t}-{\text{e}}^{-\phi t}\right)\;,$$

where $N^{\prime }=\frac{\phi }{\beta +\phi }N$. In this early stage of infection, bacterial replication occurs independently in *N* different macrophages. The mean number of bacteria per infected macrophage at time *t* is then approximated by *C*_0_(*t*)/*N*.

In the next stage of the development of the infection, we consider the fate of the *N* macrophages that phagocytosed one of the initial bacteria each. A bacterium can escape from the phagosome to the cytosol and replicate until the host macrophage succumbs to rupture and death, releasing its population of bacteria. A host macrophage's rate of rupture is proportional to the number of bacteria in its cytosol. Let *S*(*t*) be the probability a macrophage, infected at time 0, has not ruptured and died before time *t*, and consider a short time interval (*t*, *t* + Δ*t*). The probability that macrophage *i* ruptures and dies is δ *c_i_*(*t*) Δ*t*, where *c*_*i*_(*t*) is the number of bacteria in the cytosol of macrophage *i*, at time *t*. Thus, the mean number of rupture and death events from the first cohort of infected host macrophages in the time interval is *S*(*t*) δ *N*^−1^*C*_0_(*t*)Δ*t*.

A newly released bacteria may be, once again, phagocytosed by alveolar macrophages; alternatively, it may die or migrate to other parts of the body. The rates for these three events, assuming *M* ≫ *N*, are ρ *M*, μ and γ, respectively. In the first day post-infection, therefore, phagocytosis dominates: nearly all of the bacteria released by rupture and death are immediately taken up by one of the abundant resting macrophages in the alveolar space.

Let us now modify (1a) to include the immediate phagocytosis of bacteria released from the first cohort of infected macrophages. The mean number of bacteria released between *t* and *t* + Δ*t* is the mean number of ruptures in the time interval, multiplied by the mean number of bacteria released in each rupture and death event. Each of these is proportional to the mean number of cytosolic bacteria per infected macrophage at time *t*, *C*_0_(*t*)/*N*.

Let *S*(*t*) be the fraction of macrophages that survive up to time *t* after infection. Then

(3) $$\frac{\text{d}}{\text{d}t}S=-\delta \frac{C_{0}}{N}S\;.$$

**Figure 4:**
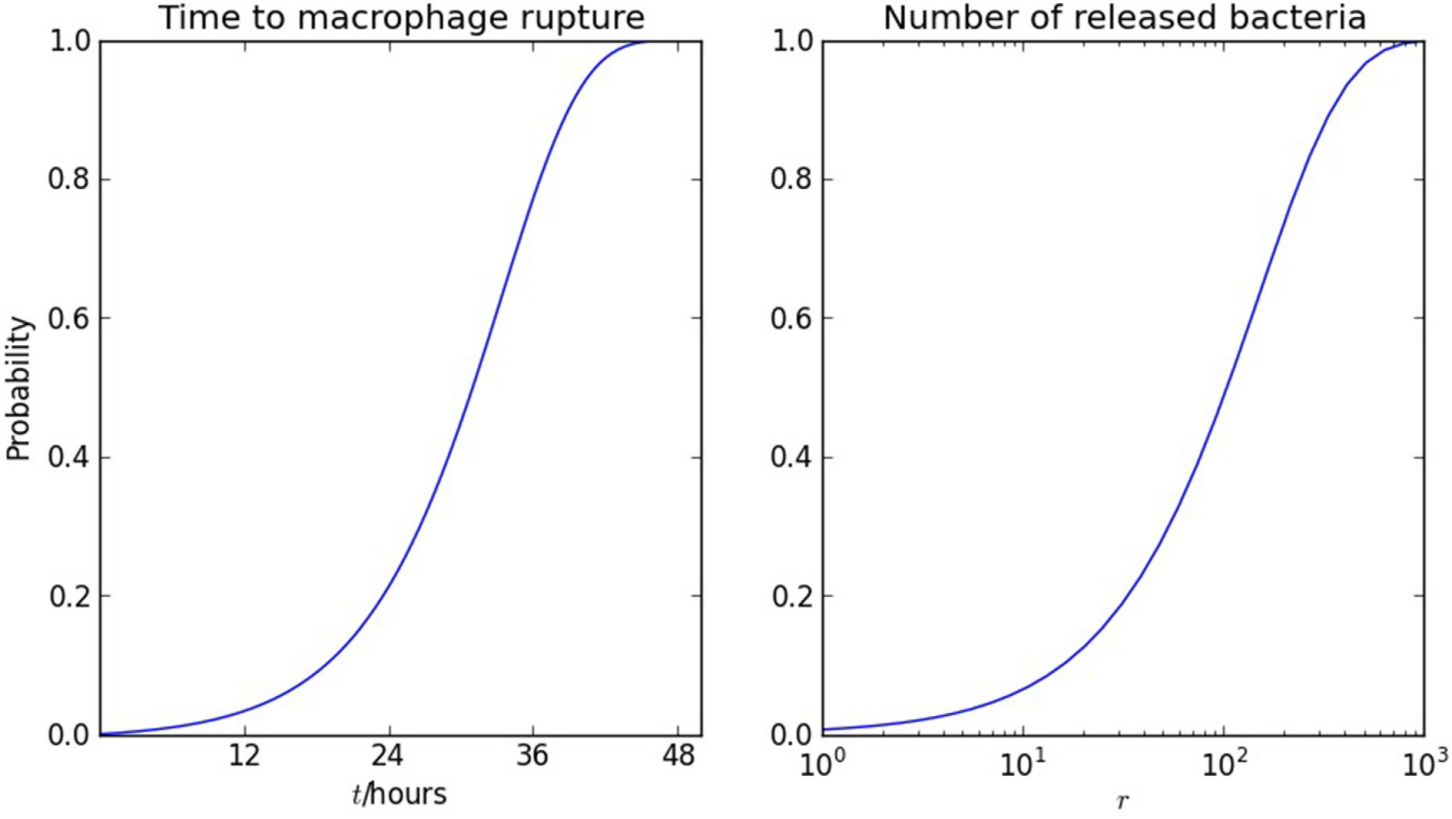
**Rupture of macrophages and release of bacteria**. On the left, the probability of macrophage rupture before *t* is 1 − *S*(*t*), where *S*(*t*) satisfies (3). On the right, the probability that the number of bacteria released from one macrophage is less than *r* is 1 − α^*r*^, using the geometric distribution (6). The parameter values are δ = 0.001 h^−1^ and β = 0.15 h^−1^.

If $\frac{C_{0}}{N}={\text{e}}^{\beta t}$, which is a valid approximation if ϕ*t* ≫ 1 and ϕ ≫ β, then $S(t)=\exp\left(-\frac{\delta }{\beta }({\text{e}}^{\beta t}-1)\right)$. The probability that a macrophage ruptures before time *t* after it is infected is plotted in Figure 4.

Let *P*_1_(*t*) be the mean total number of bacteria in phagosomes at time *t*, including the initial dose of bacteria and those released from the first cohort of infected macrophages. Then

(4) $$\frac{\text{d}}{\text{d}t}P_{1}=-\phi P_{1}+\delta \;S\;C_{0}\;\frac{C_{0}}{N}\;.$$

**Figure 5:**
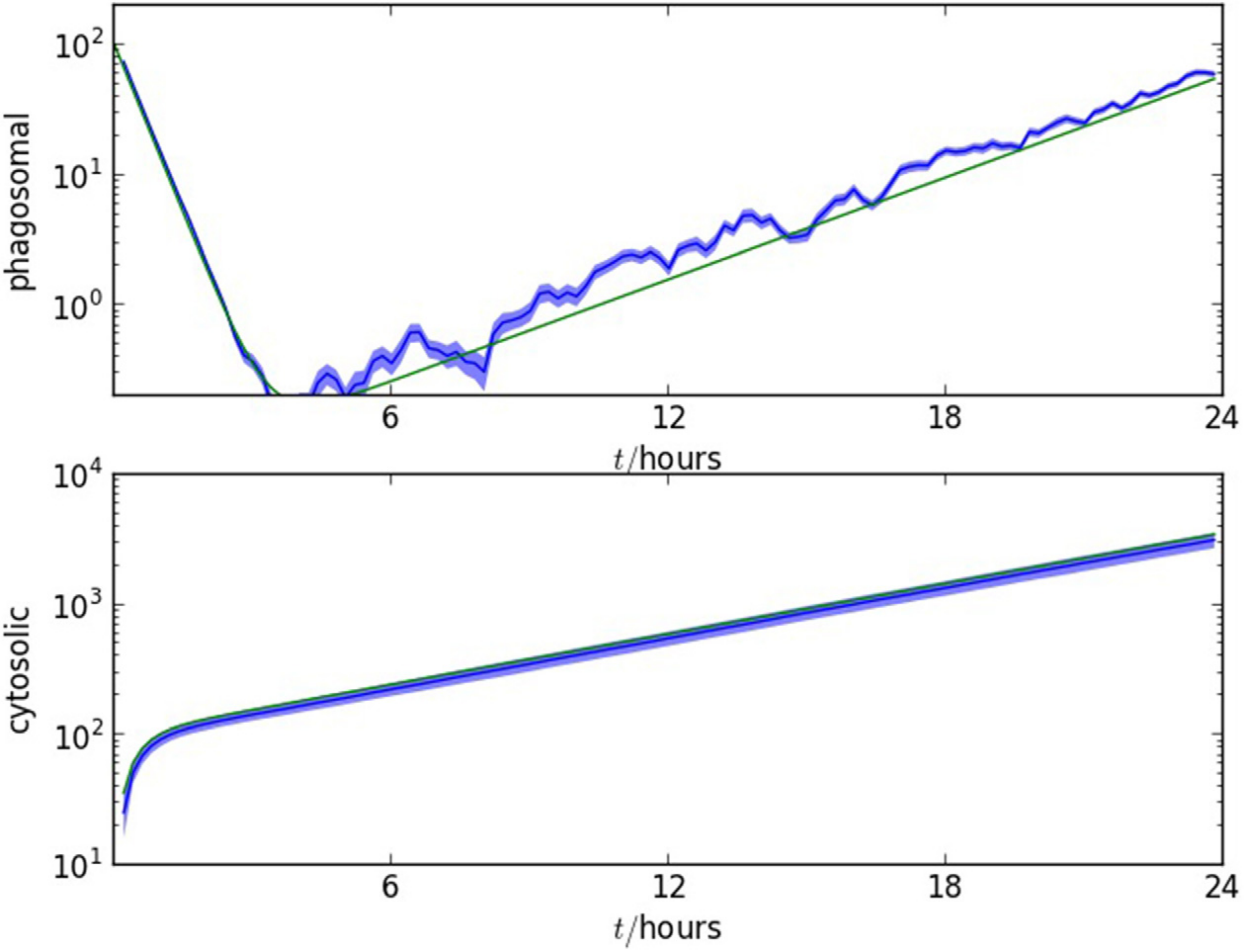
**Comparison of the stochastic model with the analytic approximations derived in section 3.2**. Upper plot: number of bacteria in macrophage phagosomes as a function of time, for the first 24 h post-infection. Lower plot: number of bacteria in macrophage cytosols as a function of time, for the first 24 h post-infection. The two figures show, in blue, one standard error range of numerical values from 10 realizations and, in green, the formulae calculated from (2b) and (4). The initial number of *F. tularensis* bacteria is Poisson distributed with mean *N* = 100. The alveolar space initially contains *M* = 10^4^ macrophages, ρ = 0.01, ϕ = 2.0, β = 0.15 μ = 0.01, γ = 0.1, ν = 0.01 and δ = 0.001. The time unit is an hour.

The solution of (4) is compared with numerical results in Figure 5. The agreement between the stochastic model and analytic approximations provide a further verification that the model is representing the infection mechanisms appropriately.

### 3.3. Distribution of bacteria released from macrophages

The model allows us to examine the dynamics from the perspective of a macrophage that is infected by a single bacterium. Once the bacterium has escaped from the phagosome, it replicates until the macrophage ruptures and dies, releasing a number of bacteria that is a random variable, *r*. When there is only one bacterium in the cytosol, the rate of division is β and the rate of rupture and death is δ. Thus, the probability that rupture and death occur before the first cell division (in which case *r* = 1) is $\frac{\delta }{\beta +\delta }$. We write

(5) $$P[r=1]=(1-\alpha )\;,\text{ where }\alpha =\frac{\beta }{\beta +\delta }\;.$$

If there are two cytosolic bacteria, the rates of division and rupture are 2β and 2δ, respectively. Thus, *P*[*r* = 2] = α(1−α). Similarly, whatever the number of bacteria in the cytosol, the probability that rupture and death of the macrophage occurs before the next bacterial division is 1 − α. The distribution of *r* is therefore geometric:

(6) $$P[r=k]=(1-\alpha )\alpha^{k\;-\;1},\text{ where }\alpha =\frac{\beta }{\beta +\delta }\;,$$

and the mean number of bacteria released when a macrophage ruptures and dies is

(7) $$𝔼(\text{r})=\frac{1}{1-\alpha }=\frac{\beta +\delta }{\delta }\;.$$

The distribution is plotted in Figure 4, together with the distribution of time to macrophage rupture, which follows directly when the doubling time of 5 h is taken into account. With δ = 0.001 h^−1^ and β = 0.15 h^−1^, 𝔼(*r*) = 151 and the standard deviation is $\sqrt{\text{var}(r)}=\sqrt{\frac{\alpha }{{\left(1-\alpha \right)}^{2}}}\;,$ comparable to 𝔼(*r*). Furthermore, with these parameter values the median number of bacteria released on macrophage rupture is 104. For comparison, the value of 358 obtained in Wood et al. (2014) by assuming that *r* is fixed and determining a best fit to human macrophage culture data, corresponds to the 91st percentile. However, the most important virtue of the theoretical expressions (6) and (7) is that they connect quantities that can be measured in independent experiments and may be targets for intervention: timescales for bacterial replication and macrophage rupture.

### 3.4. Suppression and activation of macrophages

We consider the effect of *F. tularensis* infection on the population of host phagocytes. For simplicity, we group under the heading “macrophages” alveolar macrophages and the various phagocytes of the lung, liver and spleen. Each macrophage in the model is represented as a computational object characterized by its spatial location, which does not change, state of activation and number of bacteria in phagosomes and cytosol, which do. These objects can represent any resident professional phagocytes that may be found within the alveolar space but in the majority of cases they will be alveolar macrophages. As we are considering only the early events post-infection, we do not include migration of new phagocytic cells to infected organs (Shi and Pamer, 2011). In the model, the changes that occur to the population of macrophages include changes of state, rupture and death of infected macrophages.

Macrophages are responsive to environmental changes and display a spectrum of activation states (Mosser and Edwards, 2008). We have introduced a level of phenotypic complexity to the computational objects representing macrophages. Gordon *et al*. describe five phenotypes for macrophages (Gordon and Taylor, 2005); however, we consider just three phenotypes for the purposes of the model, as follows.

The resting alveolar macrophage plays an integral role in the maintenance of the lung environment (Hussell and Bell, 2014). However, it is incapable of killing *F. tularensis*, and this is clear since a single bacterium is sufficient to cause infection. It is known that resting macrophages enter a suppressed state after ingesting *F. tularensis* and this is an integral part of the pathogenesis of the bacterium (Bosio et al., 2007).

Also, classically activated macrophages are important in clearing infection. They are able to hold infection at bay (Edwards et al., 2010), and they are the predominant emerging phenotype in the lung while the immune response begins its concerted effort to bring the infection under control (D'Elia et al., 2014). Furthermore, *in vitro* work demonstrates that bacterial numbers decline within activated macrophages (Edwards et al., 2010; Newstead et al., 2014). Therefore, we consider bacteria within activated macrophages to be removed from the model, playing no further part in the infection.

Thus, we consider three activation states that correspond to the phenotypes that play a dominant role in the early stages of *F. tularensis* pathogenesis: resting, suppressed and classically activated. While the full spectrum of activation states has not been modeled, this simplified representation of the most pertinent states for *F. tularensis* infection allows us to begin to investigate the effects of changing leukocyte phenotypes on the outcome of infection.

**Figure 6:**
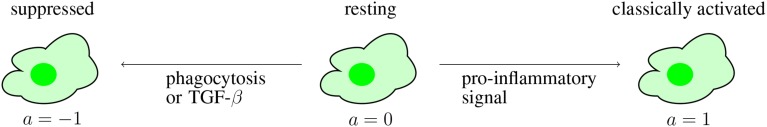
**The three states of activation of macrophages in the computational model**. Initially, all macrophages are in the resting state. During the course of infection, some pass to a suppressed state, due to phagocytosis or the effect of TGF-β. Others are activated by the effect of pro-inflammatory signals, from DAMP or IFN-γ.

In Figure 6 we illustrate the three states of activation of macrophages included in the computational model (Gordon, 2003). All macrophages are initially in the resting state, *a* = 0. A macrophage that phagocytoses a bacterium moves to the suppressed state, *a* = −1, when it is a source of anti-inflammatory signals (primarily TGF-β), that are responsible for inducing other macrophages to move to the same state. Activation, or change of macrophages to the activated state, is handled differently. Each time a macrophage ruptures and dies, inflammatory signals are released. These will include both Damage Associated Molecular Patterns and Pathogen Associated Molecular Patterns (DAMPs and PAMPs). For the purposes of our model it is assumed that these signals will affect one other macrophage in the same compartment where, if it is a resting macrophage, it becomes activated. Activated macrophages produce pro-inflammatory signals, such as interleukin IL-12, that cause lymphocytes to produce IFN-γ (Mosser, 2003; Mosser and Edwards, 2008). Activated macrophages compete for free bacteria on the same basis as resting and suppressed macrophages. Bacteria internalized into activated macrophages will either grow slower or be killed (Edwards et al., 2010). For the purposes of the model, we assume that such bacteria play no further role in the acute stage of the disease. Thus, the cytosolic bacterial load of activated macrophages is set to zero.

Thus, there are two competing processes acting to alter the macrophage population in each spatial compartment, in part the direct effect of *F. tularensis*, and in part cytokine-mediated, as follows.

#### 3.4.1. Suppression

A macrophage in the resting state (*a* = 0) that phagocytoses a bacterium or receives a TGF-β signal becomes insensitive to activation and TGF-β-producing (*a* = −1). The TGF-β produced by a macrophage in this state suppresses resting macrophages in the same spatial compartment with rate ν.

#### 3.4.2. Activation

Pro-inflammatory signals, such as IFN-γ and innate ligands released by the rupture and death of infected cells, induce a resting macrophage to become classically activated (*a* = 1) (Polsinelli et al., 1994; Gordon, 2003). Macrophages in the classically activated state are able to produce respiratory bursts and secrete pro-inflammatory cytokines. Thus, their cytosolic bacterial load is always zero. In the computational model, each macrophage rupture and death event affects one other macrophage, causing activation if it is in the resting state. Each spatial location also has an IFN-γ concentration, *G*(*t*), a real number that increases at a rate proportional to the number of activated macrophages. When *G*(*t*) exceeds a threshold, here set to the value 100, it causes resting macrophages to become activated with rate ν.

**Figure 7:**
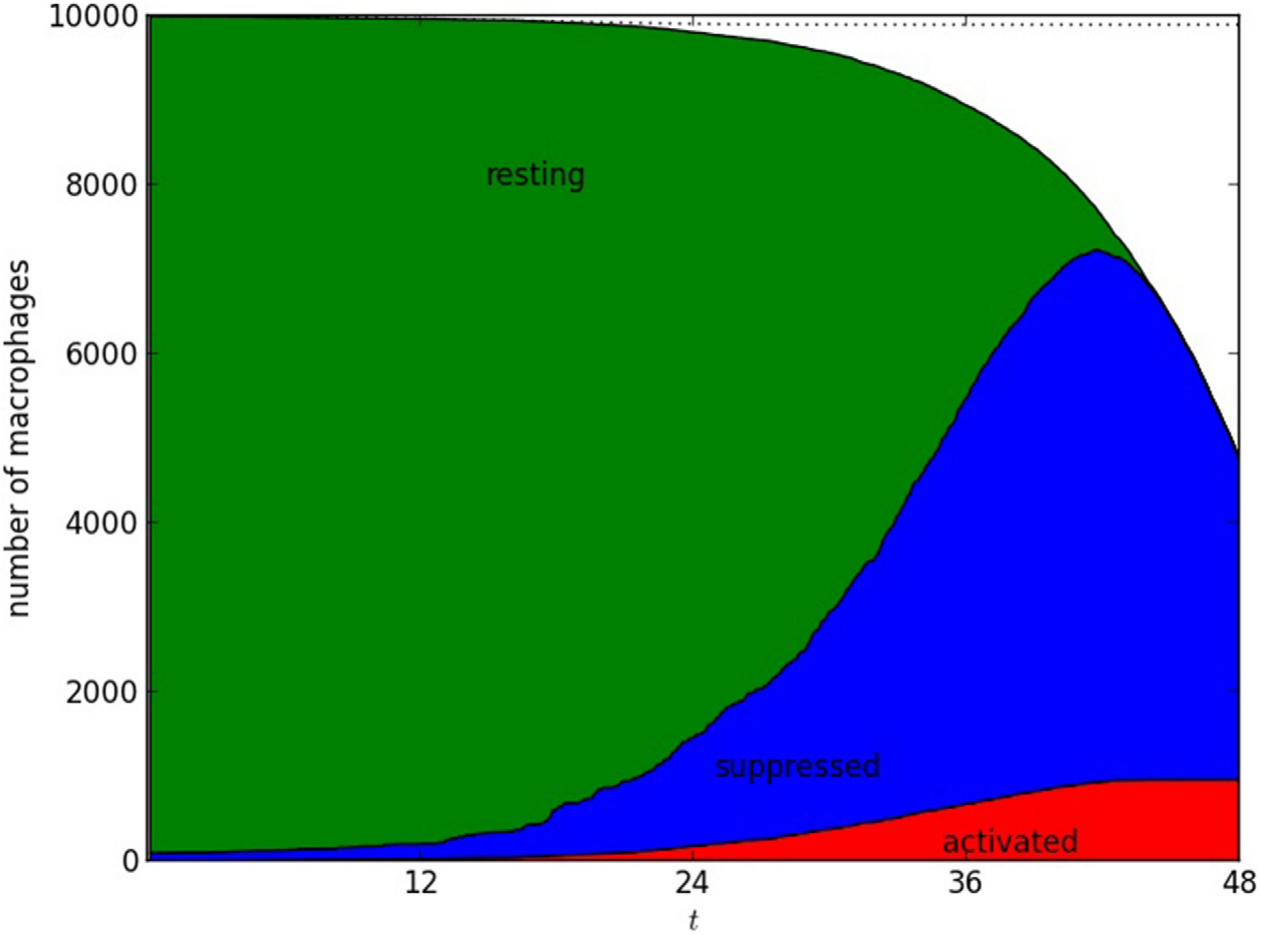
**Number of alveolar macrophages in resting, suppressed, and activated states**. One numerical realization is shown; time is measured in hours, after infection with *N* = 10^2^ bacteria. The infected macrophages, themselves in the “suppressed” state, produce TGF-β that is responsible for increasing the size of the suppressed population. The population of activated macrophages appears as a result of rupture and death of infected macrophages, and increases due to the effect of IFN-γ. The parameters used were ρ = 0.01, ϕ = 2.0, β = 0.1 μ = 0.01, γ = 0.2, ν = 0.01, δ = 0.001. The time unit is an hour.

All macrophages in each spatial location are initially resting; the *N* alveolar macrophages that phagocytose the initial dose of *F. tularensis* bacteria are immediately changed to the suppressed state. Activated macrophages begin to be found as the first cohort of infected macrophages rupture and die (see Figure 7). During the early stages of pathogenesis (up to 48 h post-infection) most of the bacteria and macrophage dynamics takes place in the lung, so that migration events to spleen or liver are negligible.

## 4. Discussion

Mechanistic understanding, from *in vivo* and *in vitro* experiments, is the basis of computational models. Within-host *in silico* models are an indispensable part of refining, replacing and reducing animal experiments. In particular, they can be used to investigate mechanisms associated with disease outcome, facilitate extrapolation from animal models to humans, guide experimentalists in designing animal studies, and encapsulate knowledge in a concrete and quantitative manner.

We present a basic stochastic model of the early stages of *F. tularensis* pathogenesis that is governed by a small number of experimentally verifiable parameters. The model includes the essential processes of macrophage infection, macrophage suppression and activation, bacterial death, phagosomal escape to the cytosol, bacterial proliferation, and macrophage death. The aim is to understand the mechanisms behind the infection process in order to inform the exploration and development of potential countermeasures. This work provides a foundation on which further complexities can be added. The model hypotheses and computations are stochastic, but the deterministic equations in Section 3.2 serve to validate our assumptions about parameter values. The model generates bacterial growth that accurately simulates *in vivo* experiments. Furthermore, we have determined probabilistic expressions to describe the time taken for infected macrophages to rupture and the expected number of intracellular bacteria released when this happens.

Pairing a computational model with pharmacokinetic data and models describing the concentration of novel antimicrobials could potentially reduce the requirement for the use of animals in research. In addition, computational models such as this can be used to estimate the level of classical macrophage activation needed to prevent infection taking hold. Model assumptions can be investigated theoretically to refine hypotheses, for instance regarding the efficacy of IFN-γ for activating macrophages. Our modeling framework also makes it possible to consider alternative scenarios, for example host cells acting as vectors transporting bacteria through the circulatory system.

We are developing a more comprehensive mathematical model of bacteria-host interaction that includes cytokines, different host phagocytes, and other arms of the immune system (Gordon and Taylor, 2005; Cowley and Elkins, 2011; Moreau and Mann, 2013). We shall also consider more organs of the body and the pattern of migration between them (Ganusov and Auerbach, 2014), motivated by the experimental data of D'Elia et al. (2013). In other organs, significant bacterial load is found from day three, along with pro-inflammatory cytokines. Immune subversion, cytosolic replication, rupture and re-uptake dominate the dynamics in the early stage, delaying proliferation. Once several rounds of macrophage rupture have occurred, there are then sufficient numbers of free bacteria to migrate to other organs, be taken up by local phagocytes, and replicate within their host cytosols. This can be modeled naturally in our framework, and leads to bacterial load profiles in other organs that emulate those observed in experiments with the BALB/c model.

The BALB/c murine model is well-characterized and there is a consistent set of data, making it a suitable starting point for computational model development. We intend to extend our methodology to consider other species of current relevance in the development of treatments, such as the rat and marmoset models of infection. When these cases are better understood, the long-term aspiration is to use the common computational framework as a means for informed extrapolation between species, ultimately to gain insight into factors that affect human tularemia and the treatment thereof.

The pathogenesis of different strains of *F. tularensis* and other infectious agents is necessarily different from that of SCHU S4 and specific models will be required for each pathogen of interest. However, our work provides a computational framework that can readily be adapted and extended to other agents, provided there is sufficient mechanistic understanding and data for parametrization. Therefore, this approach provides a basis for encapsulating and elucidating the mechanisms of infection and pathogenesis of *F. tularensis* SCHU S4, resulting in a computational tool to support practical experimentation. Models such as this may be iteratively extended and refined to incorporate new data and knowledge on host-pathogen interactions, as it is generated in the future.

## Author contributions

The model was designed by Carmen Molina-París, Joseph J. Gillard, and Grant Lythe, based on the advice and experimental models of Thomas R. Laws.

### Conflict of interest statement

The authors declare that the research was conducted in the absence of any commercial or financial relationships that could be construed as a potential conflict of interest.
